# Absolute permeability estimation from microtomography rock images through deep learning super-resolution and adversarial fine tuning

**DOI:** 10.1038/s41598-024-67367-1

**Published:** 2024-07-19

**Authors:** Júlio de Castro Vargas Fernandes, Alyne Duarte Vidal, Lizianne Carvalho Medeiros, Carlos Eduardo Menezes dos Anjos, Rodrigo Surmas, Alexandre Gonçalves Evsukoff

**Affiliations:** 1 COPPE-Federal University of Rio de Janeiro, Mailbox 68506, Rio de Janeiro, Rio de Janeiro 21941-972 Brazil; 2 CENPES-Petrobras, Av. Horácio Macedo, 950, Rio de Janeiro, Rio de Janeiro 21941-915 Brazil

**Keywords:** Energy science and technology, Engineering

## Abstract

The carbon capture and storage (CCS) process has become one of the main technologies used for mitigating greenhouse gas emissions. The success of CCS projects relies on accurate subsurface reservoir petrophysical characterization, enabling efficient storage and captured $\textrm{CO}_2$ containment. In digital rock physics, X-ray microtomography ($\upmu$-CT) is applied to characterize reservoir rocks, allowing a more assertive analysis of physical properties such as porosity and permeability, enabling better simulations of porous media flow. Estimating petrophysical properties through numeric simulations usually requires high-resolution images, which are expensive and time-inefficient to obtain with $\upmu$-CT. To address this, we propose using two deep learning models: a super-resolution model to enhance the quality of low-resolution images and a surrogate model that acts as a substitute for numerical simulations to estimate the petrophysical property of interest. A correction process inspired by generative adversarial network (GAN) adversarial training is applied. In this approach, the super-resolution model acts as a generator, creating high-resolution images, and the surrogate network acts as a discriminator. By adjusting the generator, images that correct the errors in the surrogate’s estimations are produced. The proposed method was applied to the DeePore dataset. The results shows the proposed approach improved permeability estimation overall.

## Introduction

The carbon capture and storage (CCS) process has emerged as one of the main technologies used for mitigating greenhouse gas emissions. Currently, geological formations serve as storage for $\textrm{CO}_2$. For example, sedimentary rocks that act as reservoirs of hydrocarbons have been increasingly used for $\textrm{CO}_2$ storage due to their good petrophysical properties (their high porosity and permeability) and frequent association with structural traps that guarantee containment^1^. In these cases, $\textrm{CO}_2$ is usually stored as a result of the application of enhanced oil recovery (EOR) techniques. Deep saline aquifers, found in several continental and marine sedimentary basins, are also frequently used as geological $\textrm{CO}_2$ reservoirs and have greater potential for $\textrm{CO}_2$ storage in terms of volume than do reservoir rocks in oil fields^2,3^. Petrophysical characterization provides crucial information for the successful planning, implementation and monitoring of CCS projects, making it possible to identify suitable areas for $\textrm{CO}_2$ injection and storage^4^. In general, regions with good porosity and permeability and impermeable caprocks are considered good reservoir–cap system choices since this combination of factors prevents $\textrm{CO}_2$ from migrating, thus ensuring effective and safe storage^5^.

The analysis of digital images with the aid of X-ray microtomography ($\upmu$-CT) is a powerful technique that is widely used to characterize the physical structure of porous rocks^6–9^, allowing a detailed analysis of their internal characteristics, including their porous structure. This analysis allows for faster and more assertive characterization of rock properties that are essential for understanding how $\textrm{CO}_2$ interacts with a reservoir and whether it will actually be stored, as well as estimating the amount of $\textrm{CO}_2$ that can be safely injected and retained.

Different numerical simulation models have been proposed to estimate petrophysical properties, especially the absolute permeability^10,11^. However, the accuracy and reliability of traditional numerical approaches are strongly dependent on high-resolution images of porous media, which greatly limits their implementation for engineering applications^59^. Lower resolution images, such as those of whole core samples, provide greater sample coverage and capture more sample heterogeneity, but do not adequately represent the pore structure. On the other hand, images with higher resolution permitting direct computation of fluid flow properties have correspondingly smaller fields of view. These small images, though high in resolution, only cover a representative volume for the most homogenous rocks and are very different in scale from core plugs used in conventional experiments^60^. Thus, if a permeability simulation model is calibrated on low-resolution images to achieve the same precision as a model based on high-resolution samples, it would guarantee a characterization with the same representation of permeability obtained in the laboratory, since the volume of the sample would be comparable.

Currently available imaging technologies involve an inherent trade-off between resolution and field of view (FoV)^12,13^, such that the image resolution is indirectly proportional to the sample size. A higher resolution is implied for smaller samples, which are usually ten times smaller than the standard size of plugs and lateral samples used in industry. In practice, two (or more) acquisitions are usually needed for the same sample depending on the desired resolution. This is an important issue since images need to be generated at a high enough resolution to be able to detect micrometer-sized pore features while covering a large enough region of the sample to be representative^14^. The super-resolution methodology aims to minimize this problem since there is no need to perform new acquisitions at higher resolutions to obtain satisfactory results in numerical simulations, saving time in the process.

The super-resolution problem can be defined as the problem of generating a high-resolution image from a corresponding low-resolution image. The concept of super-resolution was first discussed in^15^, where the aim was to increase the resolution of an optical system beyond the diffraction limit (the limit at which it becomes impossible to distinguish two objects). In comparison with images, one can think of the diffraction limit as the sample resolution of the image (the point where it becomes impossible to zoom in on an image without losing quality). In recent years, many advances have occurred in this area, mainly with the advent of deep learning models. Super-resolution is a challenging problem because it is an ill-posed problem with several solutions; i.e., several high-resolution images could correspond to the same low-resolution image^16^. Nevertheless, super-resolution models attempt to learn a mapping between low-resolution and high-resolution images to apply it to other low-resolution images.

The main objective of this paper is to make accurate estimations of the absolute permeability from low-resolution images. The absolute permeability was chosen as the property of interest because it provides valuable information on the pore space distribution, interporous connections, efficiency and flow pressure throughout the sample. The proposed approach uses two deep learning models: a super-resolution model that enhances the image resolution and a numerical simulation surrogate model that predicts the absolute permeability of a high-resolution image. After both models are trained, they are combined using a generative adversarial network (GAN)^17^ framework to generate better absolute permeability estimates. The surrogate model, which is kept frozen, acts as a discriminator, while the super-resolution model acts as a generator. By backpropagating the errors from the surrogate to the super-resolution model, we aim to adjust the latter parameters, i.e., adjust the high-resolution image generated, to correct the errors made by the super-resolution model and thus produce better absolute permeability estimates.

One might wonder why machine learning methods are preferred over standard numerical simulations such as Lattice Boltzmann or Stokes. The Lattice Boltzmann method is computationally expensive and primarily focused on transient flow estimation. In contrast, models like PNM and Stokes come with additional limitations. PNM heavily depends on the parameters used for generating the simulation graph, which are intrinsically linked to the segmentation process. While the Stokes model is more robust and accurate for calculating absolute permeability, it is also computationally complex. Both PNM and Stokes require high-resolution images, which are costly and time-consuming to obtain. Our method, which directly utilizes low-resolution images, avoids the need for high-resolution data. This is especially beneficial in new reservoirs, where quick estimates can optimize decision-making and reduce costs. Additionally, machine learning significantly reduces the simulation time, which can be substantial depending on the size of the rock sample.

To summarize our contributions, we (1) propose a novel three-step methodology for permeability estimation using low-resolution samples, which includes training a surrogate model for permeability estimation, training a super-resolution model, and fine-tuning the super-resolution model using errors from permeability estimation made by the surrogate model; (2) introduce a fine-tuned correction approach where the super-resolution model is adjusted based on errors backpropagated from the permeability estimates, effectively improving the accuracy of the predictions; (3) adapt the model topologies to handle 3D data using three 2D convolutional branches to maintain spatial cohesion, and merge their outputs for both super-resolution and regression purposes.

The remainder of this paper is organized as follows. Section “Related works” presents related works found in the literature. Section “Materials and methods” discusses the materials and methods used in this work. Section “Results” presents the results, and the conclusion is presented in last section.

## Related works

Recently, researchers have focused on the use of deep learning approaches to solve both the permeability estimation problem and the super-resolution problem; however, these approaches often outperform classical methods in terms of performance. As expected with deep learning algorithms, a variety of different approaches (with varying network structures, training strategies, cost functions, activation functions and so on) have already been applied to this problem, with a special focus on computer vision models such as convolutional neural networks (CNNs)^18^. The large number of works involving CNNs can be explained by the image-based nature of digital rock analysis^19^. When we consider the absolute permeability itself, the reason is even more straightforward given that the pore geometric space characterizes the absolute permeability and is thus mappable by CNNs^20^.

Considering permeability estimation, several recent works have focused on 3D CNNs with some success^21–26^. In^24^, a table comparing the results obtained by several researchers utilizing 3D CNNs was provided. The coefficient of determination ($r^2$) results ranged from 0.86 to 0.95. Overall, these works show that 3D CNNs are capable of predicting the permeability of rocks in a manner that is both fast and accurate and have provided interesting insights. In^23^, the authors showed that the introduction of physical information in the form of porosity and tortuosity constraints not only improved the network performance but also reduced the number of samples needed for efficient training but somewhat alleviated out-of-range problems. In^26^, a 3D CNN mapped the morphological structure of the pore space and provided a solution to the Navier–Stokes equation. One of the major issues with 3D CNNs (a point that we also raised) is the large number of parameters in these types of networks. To address this problem, the authors of^27^ proposed the use of a transformer-based network, merging a 2D CNN with self-attention mechanisms, to estimate the permeability, achieving an $r^2$ of 0.96. This work is especially interesting to us because the authors used the same dataset as the one we used (although we used all samples and they selected a subset of samples).

Regarding super-resolution models, CNN models are ubiquitous. Several super-resolution works have addressed both natural and tomographic images. The first use of CNNs for super-resolution was described in^28^, where the authors showed that traditional sparse-coding-based super-resolution can be viewed as a deep convolutional model. The authors employed a simple (lightweight) CNN with the mean squared error as the cost function and compared their approach with (then) state-of-the-art approaches to achieve better results. Regarding the use of deep learning in $\upmu$-CT, several interesting works have been published^29–32^ aiming to resolve the limitation of resolution in Computed Tomography (CT) scans, therefore achieving better pore and fluid-flow properties characterization.

In^31^, the authors proposed a 12-layer 3D CNN for super-resolution in microtomography. Sandstone and carbonate datasets were utilized, and the results were enhanced at various scales (x2, x3, and x4); interestingly, the networks achieved better results when trained on multiple resolutions. Bentheimer sandstone and Estaillades carbonate were used in^30^. Here, a CNN model was used for super-resolution and achieved better results (3-5 dB peak signal-to-noise ratio) than did typical interpolation methods. One of the interesting aspects of this work is that the sharpness of the edges was restored because the super-resolution methods removed high-frequency noise from the images. Hou et al.^32^ employed a GAN model constrained by prior and perceptual information to enhance the resolution of digital rock images, aiming to improve image quality and detail for advanced analysis. Their results indicated that their method outperformed conventional CNN models and interpolation methods. Ma et al.^29^ propose to enhance the resolution of CT scans using GANs to improve the predictions of simulations of fluid flow properties. They employ a standard 3D GAN super-resolution workflow to achieve high-resolution images and analyze them through pore network extraction and subsequent fluid flow simulations. They also compare the results at different resolutions to understand how decaying resolution impacts various fluid flow properties. The authors demonstrated that GANs effectively increased the image resolution, offering better estimates of pore network properties despite overestimating the porosity.

Even though our approach has a super-resolution step, that’s not our main contribution. Our objective is to improve permeability predictions by adding a surrogate model to retropropagate the error and adjust the high-resolution images generated so that the surrogate model could achieve better permeability predictions. The studies collectively display the capability of deep learning, particularly CNNs and GANs, in both permeability and super-resolution tasks. However, challenges such as correct characterization of pore space and the trade-off between resolution and sample size remain areas for further research. As stated, we aim to better characterize samples permeability by creating a adversarial-like approach to adjust enhanced images to achieve better permeability estimations.

## Materials and methods

### DeePore dataset

This work was carried out on the DeePore open dataset^33^, which contains many high-resolution (1 $\upmu$m) binarized microtomographic images. The DeePore dataset is composed of a wide range of porous materials that were created by combining naturally occurring porous textures, generating a total of 17,700 semireal 3D images with $256^3$ voxels. To do that, the DeePore dataset used a combination of Sandstone, Carbonate and Sandpack rocks. In addition to the samples themselves, the DeePore dataset also contains 30 physical properties of each sample substituted using physical simulations on the corresponding pore network models (permeability, mean throat radius, mean throat length, etc.). It is worth mentioning that the authors of DeePore have provided a Python package alongside the dataset. One of the available functionalities of this package is an outlier screening function. After running the provided function to remove outliers, 15,947 samples were included in the dataset. The package can be accessed here: https://github.com/ArashRabbani/DeePore.

### Petrophysical analysis

Petrophysical characterization provides crucial information for the successful planning, implementation and monitoring of CCS projects, making it possible to identify suitable areas for $\textrm{CO}_2$ injection and storage. In general, regions with good porosity and permeability and impermeable caprocks (rocks with low porosity and permeability), are considered good for reservoir–cap system choices since this combination of factors prevents $\textrm{CO}_2$ from migrating, thus ensuring effective and safe storage^5^.

In this article, pore network models (PNMs)^34–36^ are used to simulate the absolute permeability, and the porosity is determined by the voxel count in binarized images. The pore size distribution (PSD) is analyzed to confirm the similarities in the pore space between the images generated by the super-resolution model and the real images. The results obtained from the characterization of the sample from the DeePore dataset^33^ are considered true results and are compared with the results generated by the super-resolution technique.

#### Porosity ($\phi$)

Using porosity, it is possible to estimate the storage capacity of a geological formation, i.e., the maximum amount of $\textrm{CO}_2$ that can be safely stored underground. Identifying zones with high porosity helps to determine ideal areas for $\textrm{CO}_2$ injection since regions with high porosity generally have greater storage capacity^37^.

The analyzed porosity was determined by the voxel count in the binarized images of the DeePore dataset. A binarized image is a volume of data with a fundamental element is called a voxel segmented into two phases, the pore and matrix phases^38^. As our problem arises in the context of analyzing porous media, these phases are used in pore voxel identification (referring to the image voxels representing the pore space of the rock) and matrix voxel identification (referring to the voxels that make up the solid phase of the rock). By convention, when a two-dimensional binarized image is presented, as an illustration of the methods in this work, the value of one will be adopted for the pore voxels and zero for the matrix voxels. The porosity can thus be calculated by the simple equation:

1 $$\begin{aligned} \phi = \frac{N}{M} \end{aligned}$$

where *N* is the total number of pore voxels and *M* is the total number of matrix voxels.

#### Pore size distribution (PSD)

The characterization of pore sizes is fundamental to the success of CCS projects, as it directly impacts the effectiveness of $\textrm{CO}_2$ injection, distribution and retention in rocks^39^. Adequate pore sizes influence the effective porosity, permeability and storage capacity, which are essential elements that ensure the safety and efficiency of long-term carbon storage^40–42^.

The methods normally used to characterize the distribution of pore sizes include mercury injection capillary pressure (MICP)^43^, nuclear magnetic resonance (NMR) logging^44^ and the max ball method in digital core analysis^45^. With advances in digital rock models, several studies have been conducted using microtomography images as inputs for digital pore-size distribution analysis to quantify the sum of volumes contained in different pore sizes^10,46–48^.

In this article, the pore size distribution (PSD) was used to validate the images generated by the super-resolution technique through a quantitative analysis of the pores present in the image. This approach allows the quantitative verification of the images generated by the proposed methodology, providing objective evidence on the fidelity of the reconstruction of fine details, such as pores.

From the segmented images, it is possible to calculate the pore size distributions of both the high-resolution (HR) and super-resolution (SR) images. This calculation requires first measuring the pore diameters present in each image and creating a histogram representing the frequency of pore sizes. Finally, a comparison is made between the pore size distributions of the HR and SR images. Then, statistical methods, such as mean comparison or similarity analysis between histograms, can be employed.

#### Absolute permeability (K)

Permeability is a petrophysical property that is related to the capacity of a fluid to be transported through a porous medium and is an intrinsic property of rock that is dependent on the geometry and connectivity of the porous space. High permeabilities help in the homogeneous distribution of $\textrm{CO}_2$ in reservoirs, preventing zones of excessive accumulation or voids where $\textrm{CO}_2$ can escape and ensuring a uniform distribution in the subsoil. Likewise, regions with good permeability allow a more efficient injection rate of $\textrm{CO}_2$ into the subsoil, optimizing the capture and storage process^49^.

As previously mentioned, the absolute permeability was estimated via a PNM through digital rock models obtained from the DeePore dataset and super-resolution data. The PNM simulation involves a network of pores and throats that topologically correspond to the structure of the rock pores; these structures were extracted directly from the microcomputed tomography images^45^. By using numerical methods such as Monte Carlo simulation, finite element methods or finite differentiation methods, the fluid flow through the pore network can be simulated. Each pore and throat can be treated as a discrete element. The permeability is subsequently calculated based on the simulated flow through the pore network^50,51^. Using Darcy’s Law method, permeability can be expressed as follows:

2 $$\begin{aligned} k=\frac{\mu L Q_{0}}{A_{0}\Delta P} \end{aligned}$$

where $L$ is the length of the sample, $Q_0$ is the flow rate calculated by integrating the sample output, $A_{0}$ is the output surface area and $\Delta P$ is the pressure gradient.

Although a PNM is a widely used technique for simulating multiphase transport in porous materials, there are few open software options available for use. In this project, the OpenPNM package^52^, which was developed jointly by several research groups specializing in simulations, was used. OpenPNM is written in Python and uses the NumPy and SciPy libraries for most mathematical operations, making it easy to use and ensuring the performance needed to perform large simulations. This package was designed to be highly flexible, suitable for any application and easily customized, allowing the use of 3D models obtained through X-ray microtomography.

It is worth noticing that the Deepore dataset employs permeability measurements in pixel squared (${\hbox {px}^{2}}$) units; thus, we have retained this format for consistency. To convert ${\hbox {px}^{2}}$ to Darcy, one can multiply the ${\hbox {px}^{2}}$ value by the square of the sample’s resolution and then divide by the Darcy constant (0.9869233). This method of using ${\hbox {px}^{2}}$ units facilitates the accommodation of images with varying resolutions.

### Proposed approach

The proposed approach can be best understood in the context in which it was conceived. Suppose that we have an accurate but slow numerical method to calculate the permeability of a sample. This method requires high-resolution samples, which are expensive and time consuming. However, lower resolution images can be acquired at much lower cost.

**Figure 1:**
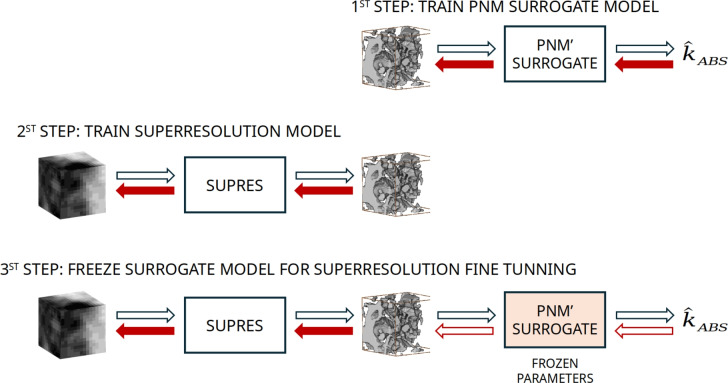
The proposed approach comprises three main steps: (1) The training of a (differentiable) surrogate model from which we can retropropagate errors. (2) The training of a super-resolution network. (3) In the proposed adversarial fine-tuning approach, the surrogate and super-resolution networks act as the discriminator and generator, respectively.

If we could generate perfect high-resolution samples, we would need to pass them only during the numerical evaluation. However, as previously explained, the super-resolution problem is an ill posed problem, meaning that while the generated samples are plausible, they are not perfect representations of the ground truth samples. Our objective here is not to achieve super-resolution per se but to achieve correct permeability estimation. Thus, the objective here is not only to generate plausible high-resolution samples but also to generate samples that lead to precise estimates. One approach is to backpropagate the errors made in the permeability estimation back to the super-resolution model to adjust the generated samples and thus yield better estimates. To backpropagate the errors, we need a differentiable module. Since the numerical computation of the permeability is not accurate, we need to generate one that is. To this end, a neural network was applied. Our method, shown in Fig. 1, is split into three steps.

In the first step, a surrogate model capable of estimating the permeability of a sample is built. In the second step, a super-resolution model is built. The novelty and the main contribution of this paper come into play in the third step. Here, a fine-tuned correction is implemented. In the standard GAN framework, two neural networks, the generator and discriminator, are trained adversarially. The objective of the generator is to produce samples that can fool the discriminator, and the objective of the discriminator is to distinguish between real samples and samples produced by the generator. During fine-tuning correction, the super-resolution model acts as the generator, generating new samples, and the surrogate model, kept frozen, acts as the discriminator. However, instead of determining the realness of a sample, the model generates an estimate of the permeability. The error generated by the difference between the actual and predicted permeabilities is backpropagated and used to fine-tune the super-resolution model.

The surrogate model receives as inputs a sample of size $256^3$ and outputs a scalar representing the permeability of that sample. The super-resolution model receives as input a low-resolution sample of size $32^3$; note that this sample was obtained by downsampling the $256^3$ sample, and a sample of size $256^3$ was output. All the networks were trained using the adaptive moment estimation (Adam) training algorithm with the mean squared error (MSE) as the cost function. Stratified cross-validation (where the order of magnitude of the permeability was used as the stratification variable) with 10 folds was used. In the training procedure, the maximum number of training rounds was set to 1000. Early stopping was applied with a patience of 30 epochs. Note that for training the super-resolution and surrogate models, the learning rate was kept constant at $1e{-}3$, while for fine-tuning correction, we employed a learning rate of $1e{-}4$. This was done to prevent the super-resolution model from “forgetting” what it had already learned.

**Figure 2:**
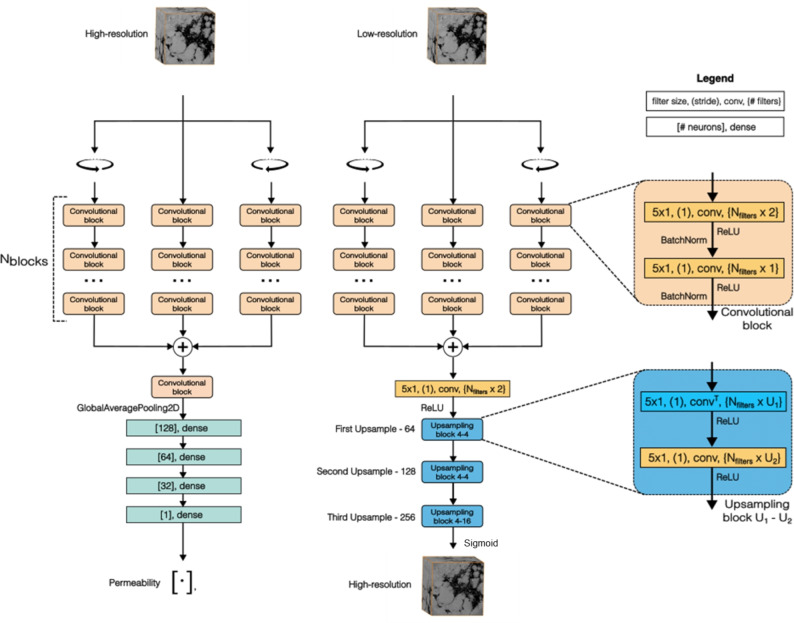
The topologies of the super-resolution and surrogate models. As shown, the models are formed mainly by convolutional blocks. Each model receives a 3D block and processes it in three different branches, each looking at a different view of the block.

The topologies for both the super-resolution and surrogate models were inspired by the generator of the SRGAN model^53^, with some modifications. The most important modification to the networks comes from the fact that the model proposed in^53^ works on 2D images while working with 3D $\upmu$-CTs. To accommodate this change, we could have changed the 2D convolutions to 3D convolutions, but this would result in a very costly model to train, given that 3D convolutions are far more computationally expensive than their 2D counterparts. However, we chose to process the 3D data because permeability is a 3D property, and super-resolution should be performed in 3D to maintain spatial cohesion. To achieve this goal, we opted to use 3 branches of 2D convolutions instead of 3D convolutions in the model. This approach was inspired by prior works that reconstructed 3D pore spaces using 2D slices (e.g.,^54–56^). Each branch processes the data from a different perspective: the first branch handles the xy-view, the second the xz-view, and the third the yz-view. This way, the model treats the 3D volume as a stack of 2D images with multiple channels, where each channel corresponds to a slice of the $\upmu$-CT scan. For instance, a $32^3$ volume is interpreted as a $32 \times 32$ image with 32 channels. To clarify the intuition behind this, in traditional 3D CNNs, the data would be processed as a single-channel 3D tensor. By contrast, the method employed reinterprets the depth dimension as channels, resulting in a 2D representation. This not only simplifies the network but also reduces the number of parameters and computational load by leveraging efficient 2D convolutions. Each branch in our model is made of 2D convolutional blocks. After the data is processed by each branch, the results are merged by summing all the branches. A final convolutional module is then applied: in the super-resolution model, this module handles image upscaling, while in the surrogate model, it acts as a regression module for permeability estimation. The model topology is illustrated in Fig. 2. Meanwhile, Table 1 provides the architectures of the two neural networks used in this study: super-resolution and permeability estimation, covering key layers, activation functions, and the number of parameters.

**Table 1:** Architecture overview of neural networks for super-resolution and permeability estimation.

Surrogate model			Superresolution model		
High resolution 3D volume ($256^3$)			Low resolution 3D volume ($32^3$)		
Rotation	–	Rotation	Rotation	–	Rotation
Block 1 - Conv2D (k = 5 $\times$ 5, s = 1, F = 32)	Block 1—Conv2D (k = 5 $\times$ 5, s = 1, F = 32)	Block 1—Conv2D (k = 5 $\times$ 5, s = 1, F = 32)	Block 1—Conv2D (k = 5 $\times$ 5, s = 1, F = 32)	Block 1—Conv2D (k = 5 $\times$ 5, s = 1, F = 32)	Block 1—Conv2D (k = 5 $\times$ 5, s = 1, F = 32)
Batch normalization			Batch normalization		
Block 1—Conv2D (k = 5 $\times$ 5, s = 1, F = 16)	Block 1—Conv2D (k = 5 $\times$ 5, s = 1, F = 16)	Block 1—Conv2D (k = 5 $\times$ 5, s = 1, F = 16)	Block 1—Conv2D (k = 5 $\times$ 5, s = 1, F = 16)	Block 1—Conv2D (k = 5 $\times$ 5, s = 1, F = 16)	Block 1—Conv2D (k = 5 $\times$ 5, s = 1, F = 16)
Batch normalization			Batch normalization		
Block 2—Conv2D (k = 5 $\times$ 5, s = 1, F = 32)	Block 2—Conv2D (k = 5 $\times$ 5, s = 1, F = 32)	Block 2—Conv2D (k = 5 $\times$ 5, s = 1, F = 32)	Block 2—Conv2D (k = 5 $\times$ 5, s = 1, F = 32)	Block 2—Conv2D (k = 5 $\times$ 5, s = 1, F = 32)	Block 2—Conv2D (k = 5 $\times$ 5, s = 1, F = 32)
Batch normalization			Batch normalization		
Block 2—Conv2D (k = 5 $\times$ 5, s = 1, F = 16)	Block 2—Conv2D (k = 5 $\times$ 5, s = 1, F = 16)	Block 2—Conv2D (k = 5 $\times$ 5, s = 1, F = 16)	Block 2—Conv2D (k = 5 $\times$ 5, s = 1, F = 16)	Block 2—Conv2D (k = 5 $\times$ 5, s = 1, F = 16)	Block 2—Conv2D (k = 5 $\times$ 5, s = 1, F = 16)
Batch normalization			Batch normalization		
Sum			Sum		
Conv2D (k = 5 $\times$ 5, s = 1, F = 32)			Conv2D (k = 5 $\times$ 5, s = 1, F = 32)		
Conv2D (k = 5 $\times$ 5, s = 1, F = 16)			Upsampling 1—Transpose Conv2D (k = 5 $\times$ 5, s = 1, F = 64)		
Global average pooling 2D			Upsampling 1—Conv2D (k = 5 $\times$ 5, s = 1, F = 64)		
Dense layer(128, ReLU)			Upsampling 2—Transpose Conv2D (k = 5 $\times$ 5, s = 1, F = 64)		
Dense layer (64, ReLU)			Upsampling 2—Conv2D (k = 5 $\times$ 5, s = 1, F = 64)		
Dense layer (32, ReLU)			Upsampling 3—Transpose Conv2D (k = 5 $\times$ 5, s = 1, F = 64)		
Dense layer (1)			Upsampling 3—Conv2D (k = 5 $\times$ 5, s = 1, F = 256)		
Linear			Sigmoid		

The implementation of the fine-tuning correction itself is very straightforward, and it basically involves creating a new model that combines both the super-resolution and surrogate models. Mathematically, we can think of super-resolution as a function *s*(*x*), where x is a low-resolution sample, and the surrogate model is a function *f*(*y*), where y is a high-resolution sample. Therefore, the combined model can be written as *f*(*s*(*x*)), where the layers that make up the *f* function are frozen; thus, only the super-resolution model is fine-tuned. This model was also trained with Adam, and the MSE served as a cost function; the only difference is that *f*(*s*(*x*)) receives as an input a $32^3$ volume and outputs a permeability estimate.

Our proposed approach was evaluated in two ways. First, to measure the results presented in this paper, permeability prediction and common metrics (such as the MSE, $r^2$ and Pearson’s correlation coefficient) were used in regression problems. Second, to measure the super-resolution itself, we explored whether the images were segmented and extracted some of the pore morphological attributes, as described in Table 2.

**Table 2:** Description of pore morphological attributes extracted from $\upmu$-CT.

Category	Attribute	Description
Pore size	Area3d	The pore boundary area accounts for the exposed surface of the external voxels
	Volume	Voxel number of the pore
Pore shape	ShapeVA3d	The 3D shape factor is defined as the pore’s sphericity
	Elongation	The ratio of the average to the largest eigenvalue of the covariance matrix
	Flatness	The ratio of the smallest to the average eigenvalue of the covariance matrix

Each of these features describes the morphological attributes of pores; thus, to calculate these attributes, the first step was to separate each pore individually. To do this, the watershed algorithm was applied. In this paper, we used the sckit-image implementation of the algorithm. The watershed algorithm basically segments each object in an image, so it is highly applicable for individualizing pores. After each pore was labelled, the features described in Table 2 were computed.

The idea here was to compare the ground truth distributions of these features with the distributions generated by super-resolution methods both before and after the proposed fine-tuning method. To test whether the distributions of these extracted features were the same, for each sample, the features were extracted, and for each feature, the Jensen divergence was computed. The Kolmogorov–Smirnov test^57^ was also applied for each feature (between the ground truth and super-resolution model results). In the end, a table of how many samples were considered to have the same distribution (i.e., the null hypothesis could not be rejected) was generated.

## Results

By applying stratified cross-validation, we ensured that the permeability distribution remained consistent across all folds. Notably, the DeePore dataset includes samples with permeabilities in the interval [6,7] $px^2$. However, due to the presence of fewer than 10 samples in this range, these samples were removed to facilitate the 10-fold cross-validation process. The training, validation and test sets used in cross-validation included 12,757, 1,595 and 1,595 samples, respectively (these numbers varied by 1 or 2 samples across the folds)

**Figure 3:**
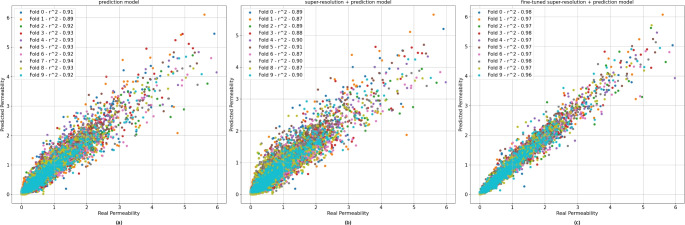
The results for each step in the proposed approach. (**a**) The surrogate model results. (**b**) The results of the surrogate model after the super-resolution model was applied. (**c**) The same results as those in (**b**) but after fine-tuning correction was applied. All permeabilities values are in $px^2$.

The overall results of the paper are summarized in Fig. 3. There are some interesting aspects to note from this figure. First, the surrogate model already achieves a high $r^2$. Second, when the super-resolution model is introduced, the $r^2$ decreases. This outcome is expected since the super-resolution images generated by the model are not perfect. Third, fine-tuning correction achieves a better result than does the base surrogate model. The explanation for this is that fine-tuning correction create images capable of correcting the surrogate model’s prediction but in the process worsens the quality of the images themselves.

The numerical results are presented in Table 3. This table shows a comparison of the performance of different models, namely, the standalone prediction model, the combination of the super-resolution and prediction models, and our proposed fine-tuning approach with both the super-resolution and prediction models. The results demonstrate a noticeable enhancement with the proposed method, as evidenced by both a higher average performance and a reduced standard deviation across metrics. As it is shown in Table 3 the results in each fold are quite similar given the small standard deviation found. Also Fig. 3 corroborates this conclusion given the similar scatter plots found across folds.

**Table 3:** Figures of merit for each model and for the proposed fine tuning approach.

	Prediction model	Super-resolution + prediction models	Fine-tuning + prediction model
MSE	0.035 ± 0.007	0.051 ± 0.007	0.013 ± 0.002
Pearson correlation	0.962 ± 0.008	0.949 ± 0.009	0.986 ± 0.002
$R^2$	0.922 ± 0.015	0.888 ± 0.015	0.972 ± 0.004

**Figure 4:**
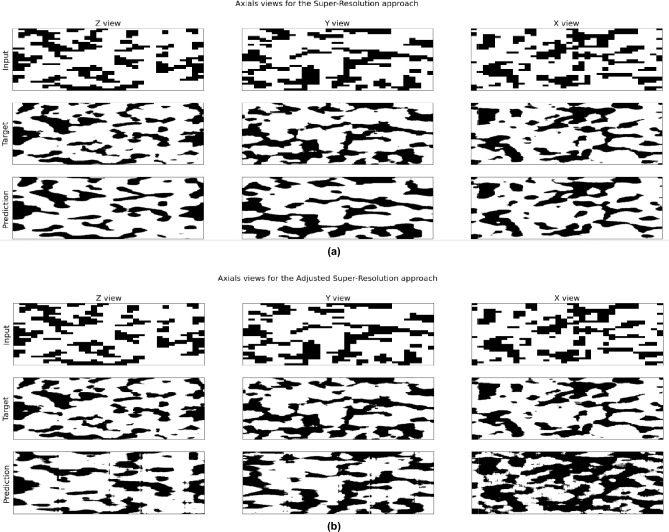
Results using the adversarial fine-tuning correction scheme. The input to the super-resolution model is shown in the first row, the ground truth views (axial, coronal, and sagittal) are displayed in the second row, and the views produced by the super-resolution model after applying the fine-tuning correction scheme are shown in the third row. Subfigures (**a**) and (**b**) show the results before and after applying the proposed fine-tuning scheme, respectively.

These results clearly indicate that the fine-tuning approach with the super-resolution and prediction models not only enhances the prediction accuracy (as shown by the lower MSE and higher $^2$) but also improves the consistency of the predictions (reflected in the lower standard deviation). To better understand the effectiveness of our method, we conducted a detailed analysis of the outputs produced by the super-resolution models. Figure 4 presents a representative sample from this analysis. An examination of these images revealed that while our approach successfully rectifies inaccuracies in permeability estimation, it introduces certain artifacts into the images as a byproduct of the correction process.

These findings present an interesting debate. Considering that every stage of the process should be faithful to physical realities, Fig. 4 highlights a shortcoming in our method. However, the ultimate goal of this paper is to accurately estimate the permeability. From this perspective, the success of our approach has been demonstrated. This is an issue that frequently occurs in practical situations and the method allows choosing the most appropriate option for each application. If the ultimate goal is prediction performance, with the high-resolution result being disposable, then artefacts do not affect the final result and the model achieves better performance. If a better high-resolution representation is desired, then a reduction in the predictive performance of the model must be considered. In the adversarial fine tuning phase, the model tuning is free to seek a super-resolution result that results in the best absolute permeability prediction. Note that, at this stage, the model attempts to fit a $32^3$-dimensional image based only on the absolute permeability error. This parameter adjustment is therefore a very difficult problem and there are numerous possible solutions (i.e., the problem is ill-posed). In this way, the adjustment algorithms make small changes to the super-resolution image that provide better performance for the surrogate model. Since the surrogate model is also a prediction model, it has no physical constraints, so changes in the super-resolution image have no physical significance. The model architecture doesn’t add the artefacts by itself since when it is used for super resolution these artefacts don’t appear.

**Figure 5:**
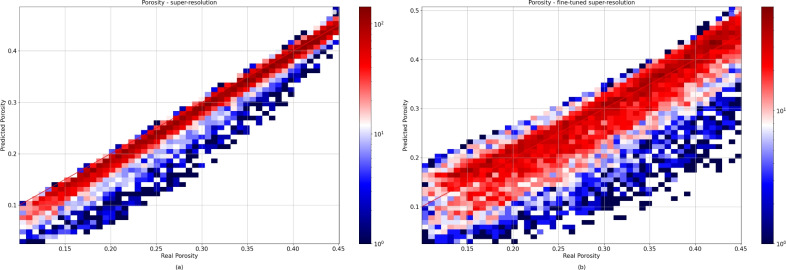
Histogram 2D depicting the porosity of the samples before (**a**) and after (**b**) applying the fine-tuning process for all test sets.

This paper primarily focuses on accurately estimating the permeability. However, we also conducted some investigations to better characterize the artifacts generated. We first studied the impact of these artifacts on the porosity of the generated samples. Figure 5 shows a comparison of the porosity values before and after applying the fine-tuning process. Initially, the porosity closely matched the ground truth, as indicated by the data points clustered near the y = x line. However, after fine-tuning, a noticeable deviation from this accuracy was observed. This trend suggests that our method compromises porosity accuracy in favor of improved permeability estimates. The introduction of artifacts, which we believe to be a result of the lack of physical constraints on neural networks, further supports this inference. These networks prioritize enhancing the permeability accuracy, disregarding adherence to physical realities in the process.

**Figure 6:**
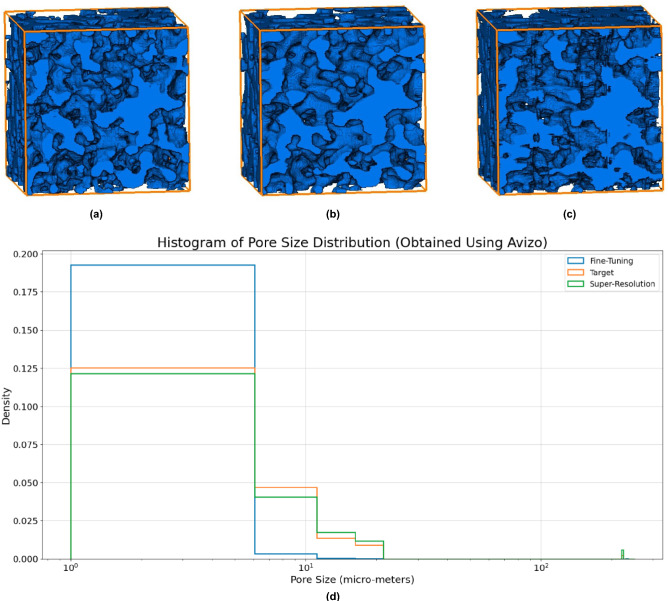
Results using the adversarial fine-tuning correction scheme in 3D illustrating: (**a**) the target image, (**b**) the image obtained through super-resolution, (**c**) the image refined using the fine-tuning method, and (**d**) the pore size distribution for each sample.

Although the artefacts are more readily observed in the 2D slices of the sample examining these samples in 3D, as depicted in Fig. 6, provides additional insights. Generally, the samples exhibit a high degree of similarity. Notably, images processed using the super-resolution method appear less detailed than their original counterparts. This smoothing effect is expected, as the input images are downscaled by a factor of eight when compared to the original data, as shown in Fig. 4a,b. This significant reduction in scale inevitably results in a loss of detail. Furthermore, images refined with the adversarial fine-tuning technique display a noticeable increase in noise. This noise can be attributed to the absence of physical constraints in the fine-tuning process. In the same image we can see the pore size (normalized) histogram for each sample. As we can see, the pore size distributions for the target and the super-resolution method are fairly similar. Meanwhile, the fine-tuning method displays a very different distribution with many more small pores.

Given the noticeable noise observed in the images processed through adversarial fine-tuning, it is also crucial to examine the morphological features of the pore structured as explained earlier. This analysis is essential as changes in the morphological distribution could indicate significant alterations in the structural integrity and functional properties of the sample. Therefore, a detailed evaluation of these properties will help in understanding the extent to which noise/artefacts introduced by processing techniques impacts the accuracy and reliability of the sample analysis. An important question that arises from these observations: Do this noisy and artifacts significantly alter the pore distributions? This issue is addressed in the following paragraph.

**Figure 7:**
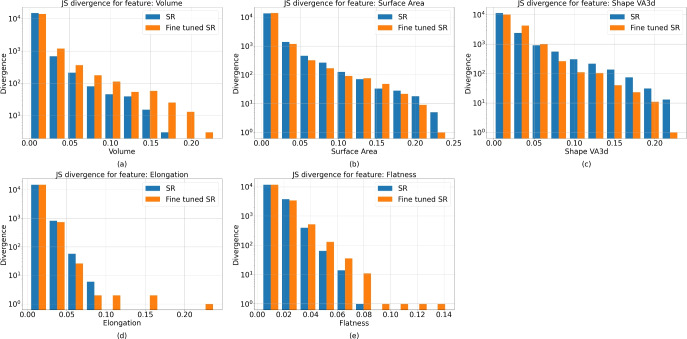
Jensen divergences for all pore morphological features (volume (**a**), surface area (**b**), shape (**c**), elongation (**d**) and flatness (**e**)) for all test sets.

Figure 7 shows the Jensen distribution for the selected features for all test sets. We found that the Jensen divergence is slightly greater for the fine-tuning approach (although the values are close to those of the base super-resolution model for most features). These results seem to indicate that even with the artifacts being generated, the images have the same overall distribution as that of the ground truth. Meanwhile, Table 4 shows the percentages of values for which the Kolmogorov test revealed that the morphological ground truth distributions of the pores were equal to those obtained for the super-resolution samples. These results support the previous results in showing that the pore distributions seem to remain close to the real ones.

**Table 4:** Percentage of the samples (average and standard deviation across folds) that the Kolmogorov–Smirnov test was not able to reject the null hypothesis for each morphological feature considered.

Approach	Volume	Surface area	Shape VA3d	Elongation	Flatness
Super-resolution	$0.964 \pm 0.027$	$0.910 \pm 0.067$	$0.891 \pm 0.082$	$1.000 \pm 0.000$	$1.000 \pm 0.000$
Fine-tuned super-resolution	$0.943 \pm 0.071$	$0.884 \pm 0.098$	$0.833 \pm 0.189$	$1.000 \pm 0.000$	$1.000 \pm 0.000$

## Comparison with other techniques

When comparing our methodologies with other techniques, we examined three distinct approaches: our baseline prediction model, the combed super-resolution and prediction model, and our fine-tuned model. Notably, the super-resolution step is tailored specifically to our problem domain, rendering direct comparisons with other methods—those without a super-resolution process—less straightforward. Additionally, the use of varied datasets across these studies necessitates a nuanced interpretation of the comparisons. These tests should be considered indicative benchmarks rather than definitive evaluations. Table 5 presents the $$R^2 $$ values for various studies employing 3D CNNs for permeability prediction, adapted from^24^.

**Table 5:** Models for permeability prediction the literature.

**Method**	**Permeability range [mD]**	**Training set size**	${{\textbf {R}}^{{\textbf {2}}}}$	Rock Type
Elmorsy et al.^24^	200–9600	> 50k	0.95	Carbonates, sandstones and limestones
Hong and Liu^25^	10–6000	< 4k	0.92	Sandstones
Kamrava et al.^22^	100–500	> 1k	0.91	Sandstones
Tembely et al.^58^	70–400	< 1k	0.87	Carbonates
Meng et al.^27^	1–156k	3500	0.97	DeePore
Current approach (surrogate model)	1–156k	12k	0.92	DeePore
Current approach (SR + surrogate model)	1–156k	12k	0.89	DeePore
Current approach (fine-tuned SR + surrogate model)	1–156k	12k	0.97	DeePore

In particular, the study by^27^ is worth noting, as it explores multiple strategies on the DeePore dataset, including 3D CNNs, physics-informed CNNs, and transformer-based CNNs. The comparison presented focuses on their transformer-based model. Our baseline models demonstrate competitive $$R^2 $$ values against those in the literature, while our optimized method attains the highest $$R^2 $$ using a less complex network (CNN versus transformer-based). It is important to highlight that despite Meng et al.’s^27^ use of the DeePore dataset, our method incorporates a larger data volume, which adds a layer of complexity to the comparison.

## Conclusions

Correctly estimating petrophysical properties is important not only for reservoir characterization aiming to exploit oil fields but also for identifying green solutions such as carbon sequestration. Many numerical simulations for the estimation of petrophysical properties require high-resolution images, which are not always available. In this paper, we address two different problems, super-resolution and permeability estimation, and combine the solutions using a fine-tuning correction scheme to obtain precise permeability estimates. Our solution improves upon the baseline solution at a low cost.

In future work, we can better characterize the artifacts that are being introduced by our solution. From our understanding, the artifacts being generated arise from the fact that our solution does not impose any physical constraints but rather only concerns the overall efficiencies. Adding physical constraints to the model, in the sense of a physics-informed neural network (PINN), could remove these artifacts while also improving the quality (and veracity) of the generated samples (both in the baseline super-resolution method and the fine-tuned version).

Other line of future work is to address the upscaling problem at the whole-core scale by employing techniques that naturally accommodate multiple scales, such as neural operators. Another possible direction is to tackle the field scale. In this case, the upscaling process would incorporate additional information, such as well logs and seismic data, in addition to data from whole-core and plug tomographic images. Our present work represents only a small part of this comprehensive process.

## Data Availability

The datasets analysed during the current study are available in the Zenodo repository, https://zenodo.org/records/3820900#.Xrnpr2hKjDc.
